# Machine Learning for Colitis-Associated Cancer in Inflammatory Bowel Disease: Evidence and Future Directions Toward Precision Medicine, a Narrative Review

**DOI:** 10.3390/ijms27135818

**Published:** 2026-06-27

**Authors:** Anna Lucia Cannarozzi, Luca Massimino, Fabrizio Bossa, Federica Ungaro, Anna Laura Pia Di Brina, Francesca Tavano, Mattia Pia Di Cosmo, Alessandra Pia Bisceglia, Maria Guerra, Monica Annese, Francesco Cocomazzi, Giuseppe Biscaglia, Silvio Danese, Anna Latiano, Orazio Palmieri

**Affiliations:** 1 Gastrointestinal Disorders Research Unit, Fondazione IRCCS—Casa Sollievo della Sofferenza, 71013 San Giovanni Rotondo, FG, Italy; a.cannarozzi@operapadrepio.it (A.L.C.);; 2 Gastroenterology and Digestive Endoscopy Department, and Division of Immunology, Transplantation and Infectious Disease, IRCCS Ospedale San Raffaele, 20132 Milano, MI, Italysdanese@hotmail.com (S.D.); 3 Division of Gastroenterology and Endoscopy, Fondazione IRCCS—Casa Sollievo della Sofferenza, 71013 San Giovanni Rotondo, FG, Italyf.cocomazzi@operapadrepio.it (F.C.); g.biscaglia@operapadrepio.it (G.B.); 4 Faculty of Medicine, Università Vita-Salute San Raffaele, 20132 Milano, MI, Italy

**Keywords:** inflammatory bowel disease, colitis-associated cancer, machine learning, artificial intelligence, dysplasia, precision medicine, biomarkers, risk stratification, surveillance

## Abstract

Colitis-associated cancer (CAC) represents a major long-term complication in patients with ulcerative colitis (UC) and Crohn’s disease (CD), the two main forms of inflammatory bowel disease (IBD). Unlike sporadic colorectal cancer, CAC develops through a distinct inflammation–dysplasia–carcinoma sequence driven by chronic inflammation and complex molecular alterations. Early detection of dysplasia and accurate risk stratification remain critical challenges in IBD management. Conventional surveillance strategies, including endoscopy, histopathology, and immunohistochemistry, are time-consuming, operator-dependent, and may fail to identify early neoplastic changes. In this context, artificial intelligence (AI), including machine learning (ML) and deep learning (DL), has emerged as a promising approach to improve lesion detection, molecular characterization, and predictive risk modeling. Early studies, though limited, suggest that AI-based approaches may enhance the identification of dysplasia and CAC, improve risk prediction, and support personalized surveillance strategies. Furthermore, the integration of multimodal data, including clinical, endoscopic, histological, and molecular features, may further improve predictive performance and enable precision medicine approaches in IBD. This review summarizes current evidence on AI and ML applications for CAC detection and risk prediction in IBD, discusses technical and clinical challenges, and highlights future directions for integrating AI into routine clinical practice to improve surveillance and clinical outcomes in patients with IBD.

## 1. Introduction

Inflammatory bowel disease (IBD) is a chronic, multifactorial disorder of the gastrointestinal tract characterized by relapsing-remitting intestinal inflammation associated with a variety of extraintestinal manifestations. It encompasses principally Crohn’s disease (CD) and ulcerative colitis (UC) [1]. The pathogenesis of IBD results from a complex interplay among genetic susceptibility, environmental and lifestyle factors, immune dysregulation, and diet-induced alterations of the gut microbiota [2]. Beyond its substantial impact on patients’ quality of life, long-standing IBD is associated with an increased risk of colorectal neoplasia [3]. Patients with IBD have an approximately 1.2- to 2.2-fold higher risk of developing colorectal cancer (CRC) compared with the general population [4,5]. Although only 1–2% of patients with IBD develop CRC, IBD-associated CRC accounts for approximately 15% of IBD-related mortality, with the highest risk observed in patients with extensive UC [6].

In this context, colitis-associated cancer (CAC) represents the prototypical inflammation-driven form of IBD-associated colorectal cancer (IBD-associated CRC) and is characterized by distinct pathogenic mechanisms, morphological features, and clinical behavior compared with sporadic CRC [7].

In contrast to sporadic CRC, CAC frequently exhibits early TP53 mutations, which may occur even in non-dysplastic inflamed mucosa. This finding reflects an inflammation-driven carcinogenic pathway rather than the classical APC-driven adenoma carcinoma sequence [8]. The increased risk of neoplasia in IBD results from a multifactorial process involving chronic inflammation, intestinal barrier dysfunction, microbiota dysbiosis, and environmental exposures in a genetically predisposed host. Among these mechanisms, sustained inflammation is considered the main driver of carcinogenesis through inflammation-induced oxidative stress, DNA damage and subsequent genomic instability. Consequently, IBD-associated CRC typically progresses through an inflammation–dysplasia–carcinoma sequence [9]. Reducing the risk of IBD-associated CRC is therefore a critical clinical objective to aimed at reducing disease-related morbidity and mortality. Colonoscopic surveillance remains the cornerstone of preventive strategies. However, lesion detection and risk stratification remain challenging due to subtle lesion morphology, field cancerization, and interobserver variability. Moreover, the effectiveness of surveillance programs in IBD has been extensively debated because of concerns regarding cost-effectiveness, resource allocation, and inconsistent long-term outcome data [10]. Although advances in endoscopic technologies and updated surveillance guidelines have improved early detection, an individualized assessment of the risk-benefit profile remains a challenge [11,12].

In parallel, emerging non-invasive molecular diagnostic strategies investigating epigenetic alterations, microRNA dysregulation, and integrative computational models are attracting increasing attention for the early detection of dysplasia and CRC, enabling more refined risk stratification [13].

The increasing availability of large-scale datasets has further accelerated interest in artificial intelligence (AI) as a tool to manage this complexity, facilitating the identification of pre-neoplastic lesions in high-risk populations, such as patients with extensive IBD or additional CRC risk factors, including smoking [14]. AI is currently being applied across a wide range of biomedical disciplines, with rapidly expanding clinical applications. In the IBD field, AI-based models are emerging as promising tools to support the analysis of disease pathophysiology, progression, and associated complications. To date, these approaches have been explored in several areas, including endoscopic image analysis, histopathological evaluation, and multiomic data integration, with the aim of improving diagnostic accuracy, disease monitoring, and therapeutic decision-making [15,16,17].

Beyond disease characterization, AI has the potential to become a cornerstone of precision medicine for CAC. By integrating clinical, endoscopic, histological, and molecular data, AI-driven approaches may optimize surveillance strategies, enable earlier detection of neoplastic lesions, and improve individualized risk stratification, ultimately improving patient outcomes and reducing disease-related mortality.

In this review, we provide a comprehensive overview of IBD-associated colorectal neoplasia and CAC, with a particular focus on the application of AI to neoplasia detection and risk assessment. We summarize the available literature on AI-based approaches in endoscopic and histologic evaluation and discuss the major challenges to their integration into routine clinical practice.

## 2. Methods

The narrative review of the literature was conducted using PubMed, Scopus, Web of Science, bioRxiv, and Google Scholar databases and included studies published between 1 January 2020 and 31 January 2026. The authors (ALC, LM, AL, and OP) independently performed the literature search, study selection, and data extraction according to a standardized protocol. Any discrepancies were resolved through discussion and consensus among the investigators. Although this was a narrative review, a structured search strategy and predefined eligibility criteria were adopted to improve transparency and reproducibility. The primary PubMed search strategy used the following search string: (“inflammatory bowel disease” AND “artificial intelligence” AND “dysplasia”). Equivalent searches based on the same keywords were subsequently performed in Scopus, Web of Science, Google Scholar, and bioRxiv.

All studies involving patients with ulcerative colitis (UC), Crohn’s disease (CD), or inflammatory bowel disease (IBD) who developed dysplasia or cancer were considered eligible, regardless of study design, number of participating centers, patient age, sample size, or type of AI strategy analyzed.

After duplicate removal, three reviewers (A.L.C., L.M., and A.L.) independently screened titles and abstracts according to predefined inclusion and exclusion criteria. Any disagreements were resolved by a fourth independent reviewer (O.P.). Studies unrelated to IBD-associated neoplasia, studies not published in English, studies without AI-based methodologies, non-human studies, editorials, commentaries, perspective articles, and review papers were excluded.

Studies were selected based on their relevance to the review topic and the reporting of key diagnostic or predictive performance metrics, including sensitivity, specificity, accuracy, recall, precision, F-score, and area under the receiver operating characteristic curve (AUC), with particular attention to the potential integration of AI systems into routine clinical practice.

The search strategy initially identified 44 records. Following abstract screening, most excluded publications consisted of narrative reviews, systematic reviews, editorials, commentaries, perspective articles, or studies not specifically addressing AI-assisted detection or prediction of dysplasia and colitis-associated cancer in patients with inflammatory bowel disease. Full-text assessment of the remaining articles resulted in the inclusion of eight original studies, which were included in the final qualitative synthesis and are summarized in Table 1.

### 2.1. Artificial Intelligence Concepts

AI is a multidisciplinary field focused on developing computational systems that can perform tasks and solve problems typically requiring human cognitive abilities and decision-making capabilities [26]. Machine learning (ML) and deep learning (DL) are two essential subsets of AI.

ML approaches include supervised, unsupervised, and reinforcement learning; however, in CAC research, supervised learning is the most commonly applied approach for prediction and classification tasks [27,28,29,30]. DL, a subset of ML, processes information through interconnected layers of computational nodes that simulate neural interactions in the human brain [31]. During training, model parameters are iteratively adjusted to detect and emphasize relevant features in the input data, optimizing predictive performance. Among DL architectures, artificial neural networks (ANNs), particularly convolutional neural networks (CNNs), have demonstrated high effectiveness in analyzing images or video frames, making them particularly suitable for applications in medical imaging and gastrointestinal endoscopy [32]. CNNs are particularly well suited for endoscopic image analysis because they can automatically learn hierarchical visual features associated with dysplasia and neoplasia, thereby facilitating the detection and characterization of precancerous lesions in patients with IBD. This capability is evidenced by the studies included in this review.

### 2.2. AI Methods for CAC: Detection, Diagnosis and Risk Stratification

AI is emerging as a promising tool for the detection, characterization, and monitoring of CAC. Chronic inflammation, mucosal remodeling, and the presence of subtle or flat lesions make early detection challenging, even for experienced endoscopists. AI-based tools have been used to automate endoscopic scoring and mucosal healing assessment, providing real-time computational decision support during procedures, as reported in the included studies [33].

AI applications are also expanding into digital pathology, where deep learning (DL) platforms can objectively quantify histological features, improving reproducibility and correlation with clinical outcomes [34]. Moreover, the integration of multi-omics data with endoscopic and histologic information, an emerging “endo-histo-omics” framework, remains an emerging research area aimed at improving multilevel risk stratification in IBD, although most approaches remain exploratory in nature and not yet externally validated [16,35,36].

The most widely applied AI approaches in this setting include the CNNs capable of analyzing endoscopic images and video frames to detect mucosal irregularities, color variations, or textural patterns suggestive of dysplasia [32] and deep ANNs, which integrate large imaging datasets, extract hierarchical features, and generate predictive outputs through multi-layered computational processing [37]; computer-aided detection (CADe) systems, which automatically highlight suspicious areas during endoscopy, improving sensitivity for subtle or flat dysplastic lesions [38]; computer-aided diagnosis (CADx) systems, which classify highlighted lesions with high specificity, aiding differentiation between benign and neoplastic tissue [39]; and hybrid and ensemble models, which combine multiple AI techniques, such as CNN-based image analysis with ML-based risk scoring, to improve diagnostic accuracy and provide comprehensive patient risk stratification in a heterogeneous set of experimental and retrospective study designs [40].

These applications were variably reported across the studies included in this review, predominantly in retrospective cohorts and feasibility reports.

## 3. Results

### AI Studies for the Detection and Characterization of IBD-Associated Cancer

Evidence for AI in CAC detection is emerging, with initial reports appearing in 2020 (Table 1). Maeda et al. [18] described the use of EndoBRAIN-EYE, an endoscopic computer-aided detection system, in a 72-year-old with an 18-year history of pancolitis, detecting two subtle sigmoid lesions confirmed as low-grade dysplasia, marking its first application in inflamed colonic mucosa. Similarly, Fukunaga et al. [19] identified a rectal lesion in a 46-year-old woman with pancolitis using endocytoscopy combined with EndoBRAIN, correctly classifying high-grade dysplasia, later treated by endoscopic submucosal dissection. These early cases represent single-patient case reports with proof-of-concept studies suggesting that AI-assisted endoscopy could be useful for UC surveillance, prompting larger studies to evaluate accuracy and reliability in IBD-associated neoplasia.

In 2022, Yamamoto et al. [20] evaluated a pilot AI model for classifying IBD-associated neoplasia and compared its performance with that of endoscopists using a deep CNN, EfficientNet-B3. The authors selected 862 non-magnified endoscopic images from 99 lesions and generated 6,375,352 images through data augmentation for AI development. Clinicopathological data, including age, sex, duration of IBD, extent of disease, tumor location, medication regimen, lesion treatment, and histological type, were obtained from medical records.

The diagnostic performance of expert and non-expert endoscopists using an AI-aided model was assessed for classifying lesions into two categories: “adenocarcinoma-high-grade dysplasia” and “low-grade dysplasia-sporadic adenoma-normal mucosa”. Images from two-thirds of the patients were used as the training, while those from the remaining one-third were used as the testing dataset. The CNN model was first pre-trained and fine-tuned on the training dataset and tested on the testing dataset. During training, the dataset was randomly divided into training and validation datasets in a ratio of 8:2 to refine the model and to optimize performance.

The image-based diagnostic ability of the AI system for two classifications of neoplasia occurring in IBD (IBDN) achieved a sensitivity of 64.5%, a specificity of 89.5%, and an accuracy of 80.6%. Meanwhile, the lesion-based diagnostic model achieved a sensitivity of 74.4%, a specificity of 85.0%, and an accuracy of 80.8%. Additionally, the image-based diagnostic accuracy of experts, non-experts, and non-experts using AI for the two IBDN classifications in the test set images was 77.8%, 75.8%, and 79.0%, respectively. Therefore, the AI model showed a modest performance improvement, particularly in non-expert-assisted settings, although differences compared with experts were limited.

Guerrero Vinsard D. et al. [21] developed a CADe system for detecting colorectal lesions in patients with IBD. The algorithm was initially trained on lesions from patients without IBD, subsequently evaluated on high-definition white-light endoscopy (HDWLE) images of IBD-associated lesions to assess baseline performance. It was then retrained to generate an IBD-CADe model using 1.266 HDWLE images and 426 dye-based chromoendoscopy images, all histologically confirmed. Eight physicians collected clinical, procedural, polyp-related, and endoscopic image data. Of the manually labeled IBD lesions, 80% were used for training, 10% for validation, and 10% for testing. The detection algorithm was based on Scaled-YOLOv4 architecture. In addition, ten short HDWLE video clips showing histologically confirmed polypoid lesions were used to evaluate performance on video sequences. The IBD-CADe system achieved an area under the curve (AUC) of 0.85 on HDWLE images, being able to detect lesions even against moderately inflamed mucosa, whereas performance was lower for chromoendoscopy images (AUC 0.65). This approach highlights susceptibility to domain shift between imaging modalities and inflammatory background variability. However, the model failed to identify pseudopolyps and generated several false positives during video analysis, indicating the need for larger image and video datasets to further improve performance.

Abdelrahim M. et al. [22] developed an IBD-specific DL model based on the RetinaNet architecture with a ResNet-101 backbone for feature extraction. The model aimed to detect and characterize lesions in 478 images from 30 patients with IBD, of whom 10 had a total of 25 neoplastic lesions, including 8 sessile serrated polyps. Lesion characterization was performed as a binary classification model (neoplastic vs. non-neoplastic). The authors also evaluated a pre-existing generic CADe model using the same validation dataset, comparing it with that of an IBD-dedicated system. The latter achieved a sensitivity of 93.5%, a specificity of 80.6%, and an AUC of 0.94. The model was further validated in real-time during endoscopic assessment of 30 consecutive patients. Among them, 11 had a total of 25 neoplastic lesions. The system achieved a lesion detection rate of 90.4%, corresponding to 4.6% lesions per colonoscopy and 0.96 neoplastic lesions per colonoscopy. The study highlighted two main challenges: reduced sensitivity due to the subtle appearance of many IBD-associated lesions and reduced specificity caused by false positive detections related to background mucosal inflammation.

A retrospective longitudinal study conducted by Hirai M. et al. [23] included 78,556 UC patients from the MHLW Japanese database, focusing on those who developed CRC during follow-up. A pointwise linear (PWL) model, an explainable ML approach that generates an individualized weight vector, was used to predict remission induction at 3 years and enable stratification. Unlike linear regression, PWL weights are derived as nonlinear functions of features, namely, clinical manifestations, response to therapy, endoscopic findings and histopathologic characteristics learned through a neural network. Considering the heterogeneity of UC, clustering was performed based on individualized weight vectors.

In the test set, the model achieved an AUC of 1.00, a recall of 0.87, a precision of 1.00, and an F-score of 0.93. Three clusters were identified: cluster 1 (colon cancer), cluster 2 (no colon or rectal cancer), and cluster 3 (rectal cancer). The analysis identified pseudopolyps and dysplasia as major CRC risk factors in UC. In addition, ML-based assessment of UC progression showed that colon cancer is more frequent in pancolitis, typically presenting in the fifth decade of life, whereas rectal cancer is more common in patients with proctitis, usually occurring in the third decade of life. These findings may support earlier CRC detection and prevention in UC. However, the ML model was developed exclusively using data from the Japanese cohort and has not been externally validated across different geographic regions, limiting its generalizability.

A transcriptomics approach was used by T. Xue et al. [24], who performed an integrated ML and bioinformatics analysis to identify cellular senescence-related genes and potential therapeutic targets involved in the progression from UC to CRC, given the pivotal role of cellular senescence in carcinogenesis. The study focused on investigating UC and CRC-associated genes linked to cellular aging, and exploring their associated genetic and transcriptional factors, as well as potential therapeutic agents.

Data were retrieved from the GEO datasets, selecting studies that included colon tissue samples from patients with UC and CRC. A total of four training sets and testing sets comprising 621 samples were analyzed. The predictive model was optimized using 10-fold cross-validation on the training set and evaluating 113 model combinations generated from 12 ML algorithms.

Five genes, namely ABCB1, CXCL1, TACC3, TGFB1, and VDR, were incorporated into the final model, each showing an AUC > 0.7 in ROC curve analysis. The combined five-gene signature demonstrated superior diagnostic performance compared with individual genes, suggesting potential utility as a biomarker panel for the colitis-to-CRC transition.

Noguchi T. et al. [25] developed an AI model using a DL algorithm to predict p53 expression directly from Hematoxylin and Eosin (H&E) stained slides. Since p53 mutations occur earlier in the dysplasia-carcinoma sequence than in the conventional adenoma-carcinoma sequence, p53 immunohistochemistry can reveal patterns associated with mutations in dysplastic lesions, aiding the detection of dysplasia and CAC.

The authors hypothesized that their CNN model could detect histomorphological patterns predictive of p53 expression on H&E slides using p53-labeled images to identify dysplastic glands. The dataset consisted of 46 paired H&E and p53-immunostained slide sets from 12 UC patients who underwent total colectomy, using 80% of patches for training, 10% for validation, and 10% for testing sets. The CNN was evaluated under classification strategies: (A) 2-class detection of p53-positive vs. negative (excluding null glands); (B) 2-class detection including null glands as positive; (C) 2-class detection including null glands as negative; and (D) 3-class detection of p53-positive, -negative, or null.

The mean average precision scores were higher than 0.73 for the analyzed tests. The model identified potential p53 mutations, enabling pathologists to perform selective immunohistochemistry and potentially facilitating the detection of subtle morphological changes. Nevertheless, the study was limited by its small cohort and the fact that multiple image patches were derived from the same patients. Overall, the AI tool outperformed conventional immunohistochemistry in terms of accuracy, cost, and time efficiency for predicting p53 expression in UC-associated dysplasia.

## 4. Discussion

CAC is one of the main complications in patients with IBD, particularly UC, developing through a distinct dysplasia-carcinoma sequence. Early detection of dysplasia and CAC is crucial, but conventional methods, such as endoscopic surveillance, histopathology, and immunohistochemistry, are time-consuming, costly, and highly operator-dependent.

Evidence from the studies analyzed and shown in Table 1 highlights that AI applications in IBD-associated colorectal neoplasia span from feasibility case reports to fully quantitative deep learning models with measurable diagnostic performance.

Early studies such as Maeda et al. [18] and Fukunaga et al. [19] demonstrated real-time feasibility of the EndoBRAIN-EYE system in detecting subtle dysplastic lesions in patients with long-standing pancolitis, although these reports were limited to single-patient case observations and should be interpreted as proof-of-concept feasibility studies.

More robust evidence is provided by Yamamoto et al. [20], who trained an EfficientNet-B3 model on 862 endoscopic images (generated from 99 lesions and over 6 million augmented samples), achieving an image-based sensitivity of 0.65 and lesion-based sensitivity of 0.74, with specificity reaching 0.90 (image-based) and 0.85 (lesion-based) and an overall accuracy of 0.81. These results highlight a moderate discriminative performance but also reflect the impact of class imbalance and dataset heterogeneity.

Similarly, Guerrero Vinsard et al. [21] evaluated a CADe system on 1266 endoscopic images (HD-WLE and chromoendoscopy), reporting an AUC of 0.85 for HDWLE and 0.65 for chromoendoscopy, demonstrating variability in performance depending on imaging modality. Abdelrahim et al. [22] further developed a RetinaNet-based DL model trained on 478 images from 30 IBD patients, achieving sensitivity of 0.93 (0.87 under real-time validation) and specificity of 0.81, with an AUC of 0.94, although limited by small sample size and potential overfitting.

Beyond image-based detection, Hirai et al. [23] applied an explainable machine learning PWL model to a large cohort of 78,556 UC patients, achieving an F-score of 0.93, a precision of 1.0, and a recall of 0.87 for CRC risk stratification, suggesting strong predictive performance although interpretation is limited by population-specific training (Japanese registry data) and lack of external validation.

In molecular applications, Xue et al. [24] evaluated 621 transcriptomic samples using 113 ML model combinations, reporting predictive performance up to ~0.7 (precision range depending on gene combination), highlighting moderate predictive power for senescence-related gene signatures in colitis-to-CRC progression. In histopathology, Noguchi et al. [25] applied CNN models to 46 paired H&E slides from 12 UC patients, reporting accuracy around 0.74–0.75 depending on the classification task (binary vs. multiclass), further emphasizing limited generalizability due to extremely small datasets.

Taken together, the studies included in this review suggest that AI may support CAC detection, lesion characterization, molecular analysis, and risk stratification in patients with IBD. DL and ML algorithms can identify subtle histological and endoscopic patterns, integrate large amounts of clinical and molecular data, and provide rapid, reproducible results, reducing operator dependency and improving diagnostic efficiency. Nevertheless, the current evidence should be interpreted cautiously, as most available studies are retrospective and have not undergone extensive external validation.

However, the clinical application of AI still faces significant limitations. Most studies rely on relatively small, often retrospective datasets, which could introduce selection bias and overestimate model performance. In addition, several image-based studies relied on extensive data augmentation strategies and relatively small numbers of unique lesions. While these approaches may improve model training, they may also increase data redundancy and the risk of overfitting, potentially leading to overly optimistic estimates of diagnostic performance. Obtaining many representative samples is particularly difficult due to the heterogeneous nature of inflammation in CAC: lesions can be subtle, non-polypoid, hidden among pseudopolyps, or located on variably inflamed mucosa, complicating the collection of large and diverse datasets. Generalizability is therefore limited, especially since datasets often come from specific centers or geographic populations. Additionally, the complexity and heterogeneity of inflamed mucosa in IBD pose major challenges that can reduce sensitivity and specificity, generating false positives and negatives, particularly in real-time colonoscopy applications. Moreover, substantial heterogeneity exists across studies in imaging modalities, patient populations, outcome definitions, and performance metrics, making direct comparisons difficult and limiting confidence in the broader applicability of reported findings.

**Figure 1:**
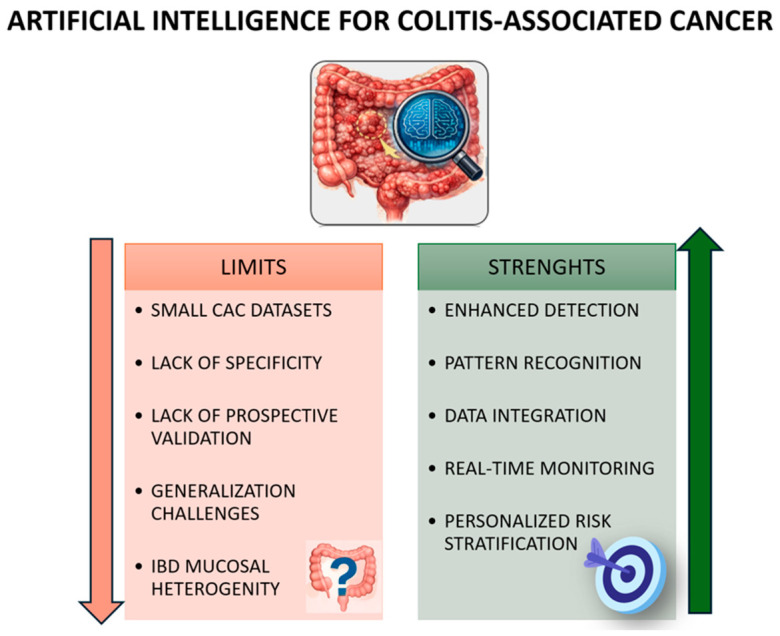
Conceptual summary of the potential strengths and challenges of applying Artificial Intelligence to the study of colitis-associated cancer.

To overcome these limitations, future developments should focus on increasing the availability and diversity of datasets, including patients from multiple centers and geographic regions, to better reflect mucosal heterogeneity and improve ML generalizability. At the same time, it will be essential to integrate multimodal data, combining endoscopic images, histology, clinical parameters, and molecular profiles, to build more complete and personalized predictive models. Models should also be tested prospectively and multicentrically, evaluating performance on new patients and in real-time colonoscopies to ensure clinical applicability. Finally, the development of explainable models and the optimization of their integration into clinical workflows will allow analysis times to be reduced without compromising diagnostic quality, reliably supporting clinician decision-making (Figure 1).

## 5. Conclusions

AI represents a promising tool to support the diagnosis and management of CAC in IBD. However, its clinical applicability remains limited by several key factors, including relatively small and heterogeneous datasets, lesion heterogeneity, limited external validation, and challenges in obtaining representative samples. Addressing these issues will require the development of more robust and generalizable models supported by prospective, multicenter, and longitudinal studies capable of capturing the full spectrum of clinical and molecular variability associated with CAC.

**Figure 2:**
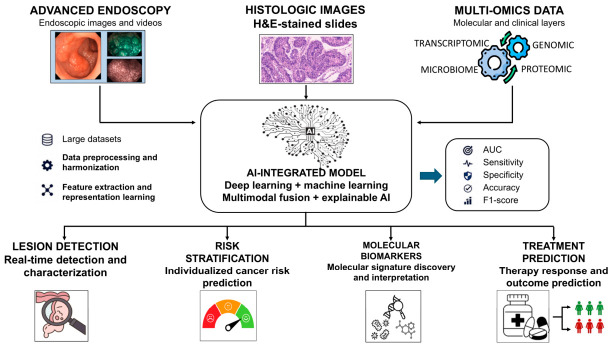
Artificial intelligence framework for colitis-associated cancer (CAC) surveillance. Schematic overview of an AI-integrated multimodal framework combining advanced endoscopy, histopathology, and multi-omics data to improve colitis-associated colorectal cancer (CAC) detection, risk stratification, biomarker discovery, and treatment prediction.

The integration of multimodal data, including clinical, endoscopic, histological, and microbiome information, represents an important future direction to improve model robustness and enhance risk stratification in patients with IBD, thereby supporting more individualized precision-medicine approaches (Figure 2). Overall, AI should currently be regarded as an adjunctive decision-support tool rather than a replacement for expert clinical judgment in the management of IBD-associated colorectal neoplasia.

## Figures and Tables

**Table 1:** Summary of key studies exploring AI models applied to IBD-associated colorectal neoplasia. ABCB1: ATP binding cassette subfamily B member 1; AI: Artificial Intelligence; AUC: Area Under the ROC Curve; CADe: Computer-aided detection; CNN: Convolutional Neural Network; CRC: Colorectal Cancer; CXCL1: C-X-C motif chemokine ligand 1; DL: Deep Learning; GEO: Gene Expression Omnibus; H&E; Hematoxylin and Eosin staining; HDWLE: High Definition White Light Endoscopy; IBD: Inflammatory Bowel Disease; MHLW: Ministry of Health, Labor and Welfare; ML: Machine Learning; PWL: pointwise linear model; TACC3: transforming acidic coiled-coil containing protein3; TGFβI: transforming growth factor beta 1; UC: Ulcerative Colitis and VDR: vitamin D receptor.

AI Studies on Analysis of IBD-Associated Colorectal Neoplasia	AI Classifier	Populations	Primary Outcomes/Clinical Results	Performance						
				Sensivity	Specifity	Accuracy	Recall	Precision	F-Score	AUC
Maeda Y. et al. [18]	EndoBRAIN-EYE	Case report: a 72-year-old man with an 18-year history of pancolitis	The system identified two subtle lesions in the sigmoid colon, both histologically confirmed as low-grade dysplasia							
Fukunaga S. et al. [19]	EndoBRAIN-EYE	Case report: a 46-year-old woman with long-standing pancolitis	The system identified a suspicious rectal lesion, classified as neoplastic and subsequently confirmed as high-grade dysplasia on histological examination							
Yamamoto et al. [20]	EfficientNet-B3	Selection of 862 non-magnified endoscopic images from 99 lesions and generation of 6,375,352 images through data augmentation	Classification of IBD-associated neoplasia	0.65 image-based; 0.74 lesion-based	0.90 image-based; 0.85 lesion-based	0.81 image-based; 0.81 lesion-based				
Guerrero Vinsard D. et al. [21]	CADe system	1266 HDWLE images and 426 dye-based chromoendoscopy images	Detection of both polypoid and non-polypoid dysplastic mucosa in patients with IBD							0.85 on HDWLE images and 0.65 on chromoendoscopy images
Abdelrahim M. et al. [22]	DL model based on the RetinaNet architecture	478 images from 30 patients with IBD	Detection and characterization of neoplastic lesions in IBD patients	0.93; 0.87 with validation in real-time during endoscopic assessment	0.81; 0.81 with validation in real-time during endoscopic assessment					0.94
Hirai M. et al. [23]	PWL model	78,556 UC patients from the MHLW database	Stratification of CRC risk in UC patients				0.87	1	0.93	1
Xue T. et al. [24]	Evaluation of 113 model combinations generated from 12 ML algorithms	621 samples from the GEO database	Identification of cellular senescence–related genes and potential therapeutic targets involved in the progression from UC to CRC					0.7 using ABCB1, CXCL1, TACC3, TGFβI, and VDR individually in the combined model, and higher with a combination of genes		
Noguchi T. et al. [25]	CNN	46 paired and p53-stained slide sets from 12 UC patients who underwent total colectomy	Prediction of p53 expression directly from H&E-stained slides					0.75 during test A (2-class detection of p53-positive vs. negative); 0.75 during test B (2-class detection including null glands as positive); 0.75 during test C (2-class detection including null glands as negative); 0.74 during test D (3-class detection of p53-positive, -negative, or null)		

## Data Availability

No new data were created or analyzed in this study. Data sharing is not applicable to this article.
